# A study of the effects of situational strength on self-efficacy and happiness: comparing individualist and collectivist cultures

**DOI:** 10.3389/fpsyg.2025.1563643

**Published:** 2025-06-27

**Authors:** Young Hyun Yeo, Ju Ho Lee, Keon-Hyung Lee

**Affiliations:** ^1^Department of Public Administration/Digital Contents, Sun Moon University, Asan, Republic of Korea; ^2^National HUSS Consortium Center, Sun Moon University, Asan, Republic of Korea; ^3^Askew School of Public Administration and Policy, College of Social Sciences and Public Policy, Florida State University, Tallahassee, FL, United States

**Keywords:** situational strength, self-efficacy, happiness, cultural influence, individualism, collectivism

## Abstract

When individual values, attitudes, and behaviors do not align with dominant cultural expectations, organizational societies often employ situational strength to promote behavioral conformity. While this may enhance organizational efficiency by minimizing variability in individual performance, it can also suppress self-expression and elevate stress—particularly for individuals in collectivist cultures who face stronger normative control. Notably, countries such as South Korea and Japan report lower average levels of happiness compared to Germany and Finland, despite comparable levels of economic development. This study investigates the psychological mechanisms underlying this disparity by examining the role of situational strength within cultural contexts. Using survey data from 608 participants across South Korea, Japan, Finland, and Germany, this study explores how perceptions of situational strength influence self-efficacy and happiness across different cultural orientations. The results indicate that situational strength significantly reduces both self-efficacy and happiness, with particularly strong effects in collectivist societies. Moreover, self-efficacy partially mediates the relationship between situational stress and happiness, highlighting its critical psychological function. Individuals in collectivist cultures experience higher levels of situational strength due to greater societal and organizational pressure to conform, whereas those in individualist cultures report higher autonomy, reduced stress, and greater psychological well-being. These findings advance situational strength theory by demonstrating that its effects on psychological outcomes are not culturally neutral. Rather, they are shaped by sociocultural environments that modulate the experience of conformity pressure. This study contributes to cross-cultural psychology by clarifying how cultural values and institutional norms interact to influence emotional and motivational outcomes.

## 1 Introduction

In any society, there is a mismatch between the whole and its parts. While societies pursue their unique cultures and values, organizations guide their members in alignment with their mission and vision. These organizations enforce explicit and implicit forms of normative control to ensure their members reflect shared values or behaviors. Ideally, when individuals naturally align their values and behaviors with societal norms, there is no conflict between the collective and its members. However, individual differences in values, abilities, and roles often lead to misalignment, resulting in varying levels of cohesion and disparity between the collective and the individual. In such cases, societies or organizations exert various tangible and intangible influences on individuals to promote harmony and achieve collective goals. These influences are called institutional forces or pressures and may be coercive, mimetic, or normative.

According to situational strength theory, environments with clear behavioral expectations and constraints can guide or restrict individual actions. In these situations, the influence of personal traits on behavior tends to diminish, and internal drivers such as self-efficacy may similarly lose their predictive power. When strong, situational strength can standardize individual behaviors by providing situational cues; when weak, behavior is more likely to be influenced by personality traits (Alam et al., 2025; OECD, 2024). The cultural characteristics of strong situations not only constrain self-expression (Gelfand et al., 2011) but also help moderate the relationship between individual differences and adaptive performance (Shoss et al., 2012).

Collectivism may inhibit or replace the formation of self-efficacy through culturally embedded social mechanisms (Meyer et al., 2010). Conversely, other research suggests that collectivism can interact with self-efficacy to enhance social or group efficacy. For example, Ajiboye and Olubela (2020) found that individuals with high self-efficacy are less prone to social loafing, while those with stronger collectivist orientations are more likely to avoid personal responsibility than assume collective accountability. These effects, however, vary across cultural contexts. In horizontal collectivist cultures—such as among East Asian youth—self-efficacy may operate in conjunction with communal responsibility to enhance social influence (Yu and Sun, 2024). These findings indicate that the relationship between collectivism and self-efficacy is not linear but contingent on cultural typologies and institutional environments.

While prior research on situational strength theory has largely emphasized its beneficial role in promoting organizational goals—by minimizing the influence of individual personality traits and standardizing job performance—there has been limited focus on its implications for personal happiness and self-efficacy. In collectivist societies, high-performing individuals may experience enhanced self-efficacy through social support. However, lower-performing individuals in such societies may suffer adverse psychological consequences that undermine both self-efficacy and happiness (Dalal et al., 2020). Cultural variations in the perception and interpretation of situational strength influence behavioral responses, situation-actor fit, and how individuals experience contextual cues. Traditional approaches to situational strength theory have centered on standardization and performance control, overlooking emotional dimensions such as self-efficacy and happiness. This study diverges from such perspectives by exploring how situational strength affects emotional outcomes, specifically self-efficacy and happiness.

The primary aim of this study is to investigate how cultural orientation—specifically individualism versus collectivism—influences the relationships among situational strength stress, self-efficacy, and happiness. Using data from four economically comparable nations (South Korea, Japan, Finland, and Germany), this study examines (a) the extent to which situational strength within organizational and societal contexts generates psychological stress, (b) how such stress affects self-efficacy and, subsequently, happiness, and (c) how these associations vary across cultures and individual-level socio-demographic. By identifying culturally contingent pathways, this research extends situational strength theory to encompass emotional and behavioral outcomes across diverse cultural settings.

This research contributes to situational strength theory by addressing cultural differences in individual behavioral responses—dimensions relatively overlooked in traditional approaches.

### 1.1 The present study

The selection of South Korea, Japan, Finland, and Germany was guided by their comparable levels of economic development despite substantial differences in quality of life and happiness. According to OECD data, these countries vary notably in demographic profiles, family structures, employment conditions, income inequality, and life satisfaction, despite similarities in education levels.

As of 2022, South Korea had 17.5% of its population aged 65+, a high tertiary education rate (69.3%), low unemployment (2.7%), and below-average life satisfaction (5.9). Japan, the oldest OECD society (29.4% aged 65+), had a 63.8% tertiary education rate and moderate life satisfaction (6.1), alongside low marriage and fertility rates. Finland, though reporting a relatively high unemployment rate (7.2%) and a moderate tertiary education rate (42.0%), ranked highest in life satisfaction (7.9), reflecting a liberal family culture with high rates of cohabitation and divorce. Germany had 22.0% aged 65+, a 32.4% tertiary education rate, and a life satisfaction score of 7.1. Its growing share of cohabiting and unmarried households aligns with multicultural and individualist trends.

This study investigates the impact of situational-strength-related stress on self-efficacy and individual happiness across four countries—South Korea, Japan, Finland, and Germany—that share similar levels of economic development. The research examines how individuals from diverse cultural backgrounds perceive and experience situational demands and how cultural differences influence the interpretation and psychological outcomes of those situations (Gardiner et al., 2020). Evaluating the appropriateness of situational strength within cultural contexts is essential for understanding individual cognition and behavior (Meyer et al., 2010). This study contributes to bridging a gap in situational strength theory by highlighting the importance of cross-cultural comparisons between cultural context and individual responses—an area that has received limited attention in prior research.

**Figure 1:**
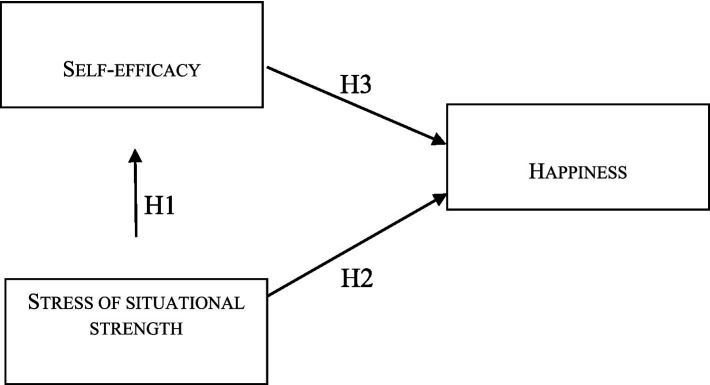
Analytical model of the study.

Based on previous research, we developed three hypotheses and the analytical model for this study (Figure 1):

*H1*: Stress arising from situational strength, designed to harmonize the collective and its components, influences individual self-efficacy.

*H2*: Stress stemming from strong situational strength impacts happiness.

*H3*: A decline in individual self-efficacy negatively affects happiness.

## 2 Theoretical background

### 2.1 Individualism and collectivism

Individualism and collectivism are essential concepts for understanding cultural differences (Heine et al., 2008; Hofstede, 1980a; Oyserman et al., 2002; Yamaguchi, 2001). These cultural dimensions are often examined in relation to socioeconomic structures (Greenfield, 2009; Inglehart and Baker, 2000) and lifestyle trends in modern societies (Talhelm et al., 2014). Hofstede (1980a: 148–170) described individualism as a cultural pattern prioritizing personal autonomy and independence, while collectivism emphasizes group harmony and interdependence. Triandis (1995) elaborated on this distinction, explaining that individualism focuses on self-reliance and individual rights, whereas collectivism values social cohesion and group harmony.

This study analyzes how situational strength and self-efficacy influence happiness among organizational members within both collectivist and individualist cultural contexts. South Korea and Japan are widely recognized as collectivist societies that emphasize group harmony, adherence to social norms, and relational identity. In contrast, Germany and Finland exemplify individualist cultures that value personal autonomy, self-expression, and independence. According to Gelfand et al. (2011), South Korea and Japan are considered “tight cultures,” characterized by strong social norms and low tolerance for deviance. Conversely, Germany and Finland represent “loose cultures,” which exhibit greater behavioral flexibility and a higher tolerance for nonconformity.

Hofstede (1997) argued that a nation’s wealth is generally positively correlated with its level of individualism. However, South Korea and Japan, despite being economically developed, maintain distinctly collectivist cultural patterns. This apparent deviation is largely attributed to the influence of Confucian heritage in East Asian societies, which emphasizes group values, social stability, and hierarchical order. Although Hofstede’s (1997) indices remain foundational, they reflect categorizations from nearly three decades ago. More recent frameworks, such as those proposed by Minkov and Kaasa (2022), offer updated indices derived from items in the World Values Survey (WVS). These updated models incorporate religiosity as a meaningful variable.

Building on Hofstede’s foundational model, Minkov and Kaasa (2022) proposed a revised individualism–collectivism (IDV-COLL) index, which incorporates religiosity as a key indicator. According to Minkov and Kaasa (2022), religious individuals often uphold fixed moral principles that guide behavior across contexts, which support a culture of behavioral consistency and ideological stability. Therefore, religiosity functions not merely as a belief system but as a cultural marker of inflexible self-concept and value preservation. Based on this updated framework, Germany (102) and Finland (88) are classified among the most individualist countries, whereas Japan (42) and South Korea (25) remain on the collectivist side of the cultural spectrum.

Minkov and Kaasa’s (2022) findings indicate a significant divergence from Hofstede’s earlier results concerning the United States (33), a discrepancy that may be attributed to the country’s unique religious landscape—particularly the collective orientation inherent in American Protestant traditions. Although the United States is commonly regarded as a prototypical liberal Western society, its IDV-COLL score suggests it does not occupy the extreme high end of the individualism spectrum, especially when compared to major East Asian countries.

The four countries examined in this study—Germany, Finland, Japan, and South Korea—were consistently found in both studies to be positioned along the individualism–collectivism spectrum in that order.

In collectivist cultures, when individual values and behaviors clash with societal or organizational norms, the pressure to conform is stronger than in individualist cultures. Collectivistic societies prioritize the collective over the individual, often exerting significant situational control to maintain cohesion. This control may heighten social tension, as individuals are expected to align their actions closely with group norms and suppress personal desires in favor of collective goals (Hofstede, 1980a; Kashima et al., 1995; Shweder and Bourne, 1982; Triandis, 1995; Triandis et al., 1988).

In organizations, the distinction between individualism and collectivism affects both the control exerted on members and the pressures they experience. Individualist cultures prioritize personal performance and accountability, often making individuals feel solely responsible for outcomes (Alvesson, 2002). Conversely, collectivist cultures emphasize teamwork and harmony, with organizational control relying on group consensus. When individual behaviors deviate from organizational norms in collectivist contexts, individuals face increased situational pressure and stress because conformity is critical (Alaybek et al., 2017; Cho and Park, 2013; Hofstede, 1980a, 1980b). A study by Gardiner et al. (2020) exploring the role of situational strength across different cultural systems suggested that it may have more impact on individual behavior within collectivist cultures (see also Harrington and Gelfand, 2014).

### 2.2 Stress of situational strength and self-efficacy

Situational strength theory (Mischel, 1977) posits that environmental cues and expectations constrain individual behavior by promoting norm adherence. Strong situations—characterized by clarity, consistency, constraints, and consequences (Meyer et al., 2010)—reduce behavioral variability and inhibit the expression of personal traits (Alaybek et al., 2017; Freudenstein et al., 2024; Yang et al., 2022).

The degree of situational strength is shaped by cultural context. Collectivist cultures tend to reinforce hierarchical authority, shared accountability, and collective decision-making, increasing external pressure on individuals to conform (Hofstede, 2001; Singelis, 1994). South Korea, for instance, exhibits persistent cultural rigidity despite its industrialization, combining strong societal norms with intense competition. This alignment fosters role overload and psychological strain when personal values or abilities diverge from societal or organizational expectations (Chen et al., 2016; French and Caplan, 1972; Karasek and Theorell, 1990).

At the organizational level, heightened situational strength may manifest as social isolation or workplace bullying, especially in cultures that emphasize conformity. Such dynamics stem from the mismatch between individual identity and collective expectations, where the pressure to align with rigid social norms increases stress and undermines self-efficacy (Björkqvist et al., 1994; Einarsen et al., 1994).

### 2.3 Self-efficacy

Self-efficacy refers to one’s belief in the ability to organize and execute actions to achieve specific outcomes (Bandura, 1977, 1997). It includes confidence, self-regulation, causal attribution, and a preference for moderately challenging tasks (Sherer and Adams, 1983; Bandura and Cervone, 1986; Locke and Latham, 1990; Zimmerman et al., 1992). These components shape how individuals cope with stress, persist in achieving goals, and regulate their emotions (Maddux, 2016; Schunk and Pajares, 2005).

In environments characterized by strong situational demands, perceived self-efficacy acts as a buffer, enabling individuals to maintain a sense of agency and emotional stability. Particularly in collectivist settings—where high levels of social cohesion and normative expectations prevail—self-efficacy plays a critical role in offsetting psychological stress (Pákozdy et al., 2024; van Zyl and Dhurup, 2018).

However, self-efficacy itself is culturally embedded. In collectivist societies, where organizational norms often override individual values, misalignment may diminish self-efficacy and, by extension, reduce happiness (Hofstede et al., 2010; Smith and Bond, 2019; Norris and Inglehart, 2019). This study aims to examine how self-efficacy mediates the relationship between situational strength and happiness across different cultural contexts.

### 2.4 Happiness

Happiness is defined as the subjective evaluation of life satisfaction, emotional well-being, and sense of purpose (Diener, 1984; Seligman, 2011). It encompasses both hedonic dimensions—such as the frequency of positive affect and the absence of negative affect—and eudaimonic dimensions, including optimism and flourishing (OECD, 2013; De Neve et al., 2013).

Rather than a fleeting emotional state, happiness is considered a comprehensive psychological indicator of life quality. It is shaped by both external structures and internal resources. Strong situational environments may erode perceived autonomy and control, thereby decreasing self-efficacy and elevating stress (Mischel, 1977; Bandura, 1997). Conversely, individuals with high self-efficacy are more likely to maintain positive affect and life satisfaction even under pressure (Schunk and Pajares, 2005; Kılıç et al., 2013).

Although numerous studies have independently examined self-efficacy and happiness, few have analyzed their interrelationship within the context of situational strength and cross-cultural variation. This study addresses that gap by exploring how situational demands and individual resources interact to influence happiness across distinct cultural settings.

## 3 Data and methods

### 3.1 Study design

This study examined how situational strength—conceptualized as perceived stress—affects happiness across four culturally distinct yet economically comparable countries: South Korea, Japan, Finland, and Germany. Data were collected over a two-year period (June 2022 to June 2024) using a mixed-mode approach. Approximately 80% of responses were gathered through in-person surveys during field visits, while the remaining 20% were collected online.

Survey implementation varied by country. In Germany and Finland, data collection was conducted in collaboration with OPIMKOTI, the Korea–Finland Education Research Center. In Japan, surveys were administered with support from a cultural anthropology professor at Soonchunhyang University, an alumnus of the University of Tokyo. In South Korea, a statistics professor from Sun Moon University advised on survey administration. Urban areas were selected as survey sites: Hamburg (Germany), Helsinki (Finland), Tokyo (Japan), and Seoul (South Korea).

To enhance cross-cultural comparability, validated items from prior international surveys were used, with particular attention to measures of situational strength, self-efficacy, and happiness. Happiness items were adapted from United Nations global well-being instruments. Survey instruments were translated by Korean professors residing in Germany, Japan, and Finland, and subsequently reviewed by local scholars to minimize misinterpretation due to linguistic or cultural nuances.

This study was supported by the National Research Foundation of Korea and complied with the Republic of Korea’s Personal Information Protection Act and the OECD Privacy Guidelines to ensure ethical data collection and participant confidentiality.

### 3.2 Participants and recruitment

Participants were recruited from local communities in each city with the support of academic collaborators. Recruitment was conducted through announcements shared via community organizations, universities, and local social networks. Participation was voluntary, and all respondents were informed of the study’s purpose and confidentiality procedures prior to completing the survey. To standardize responses related to organizational context, participants were instructed to consider their primary affiliation—such as their workplace, school, or volunteer group—when responding to relevant items.

A total of 628 responses were collected: 191 from South Korea, 103 from Japan, 183 from Germany, and 151 from Finland. After excluding cases with missing data, 608 valid responses (96.8%) were retained for analysis. Descriptive statistics for the final sample are presented in Table 1. A convenience sampling strategy was used, combining academic networks and community-based outreach coordinated by local partners.

**Table 1:** Descriptive statistics.

Variable		South Korea		Japan		Finland		Germany		Total	
		N	%	*N*	%	*N*	%	*N*	%	*N*	%
Gender	Male	98	51.3	47	45.6	49	32.5	79	48.5	273	44.9
	Female	93	48.7	56	54.4	102	67.5	84	51.5	335	55.1
Age	20–29	59	30.9	19	18.4	61	40.4	79	48.5	218	35.9
	30–39	41	21.5	7	6.8	37	24.5	59	36.2	144	23.7
	40–49	46	24.1	12	11.7	23	15.2	9	5.5	90	14.8
	50–59	32	16.8	33	32.0	18	11.9	11	6.7	94	15.5
	60–69	13	6.8	27	26.2	7	4.6	1	0.6	48	7.9
	> 70	0	0.0	5	4.9	5	3.3	4	2.5	14	2.3
Marital status	Single	109	57.1	32	31.1	90	59.6	124	76.1	355	58.4
	Married	82	42.9	57	55.3	43	28.5	34	20.9	216	35.5
	Divorced	0	0.0	11	10.7	18	11.9	5	3.1	34	5.6
	Widowed	0	0.0	3	2.9	0	0.0	0	0.0	3	0.5
Employment status	Employed	145	75.9	77	74.8	119	78.8	132	81.0	473	77.8
	Unemployed	46	24.1	26	25.2	32	21.2	31	19.0	135	22.2
Education	Middle School	0	0.0	3	2.9	12	7.9	1	0.6	16	2.6
	High School	46	24.1	37	35.9	45	29.8	22	13.5	150	24.7
	Associate’s	16	8.4	18	17.5	30	19.9	38	23.3	102	16.8
	Bachelor’s	95	49.7	37	35.9	62	41.1	71	43.6	265	43.6
	Graduate	34	17.8	8	7.8	2	1.3	31	19.0	75	12.3
Income level	1 (low)	28	14.7	60	58.3	0	0.0	44	27.0	132	21.7
	2	58	30.4	11	10.7	56	37.1	53	32.5	178	29.3
	3	76	39.8	13	12.6	33	21.9	30	18.4	152	25.0
	4	23	12.0	11	10.7	21	13.9	17	10.4	72	11.8
	5	4	2.1	1	1.0	41	27.2	7	4.3	53	8.7
	6 (high)	2	1.0	7	6.8	0	0.0	12	7.4	21	3.5
Sample (*N*)		191	31.4	103	16.9	151	24.8	163	26.8	608	100.0

### 3.3 Measures

Self-report measures were used to assess situational strength stress, self-efficacy, and happiness. All scales were drawn from validated international sources, adapted for cross-cultural use through forward–backward translation, and pre-tested for clarity. Each construct was operationalized as a composite score from multiple Likert-type items.

#### 3.3.1 Situational strength stress

This construct was measured using four items adapted from stress and cultural norm studies (Cohen and Wills, 1985; Leifels and Bowen, 2021; Gelfand et al., 2011). Items assessed organizational control, normative pressure, skill demands, and value misalignment stress. Responses were rated on a 5-point Likert scale (1 = strongly disagree to 5 = strongly agree), and the mean score formed the composite index.

#### 3.3.2 Self-efficacy

Three items were adapted from general self-efficacy and motivation research (Bandura and Cervone, 1986; Dweck and Leggett, 1988; Locke and Latham, 1990). Items assessed task confidence, persistence, and challenge preference. Responses were rated on a 5-point Likert scale, and the average formed the composite score.

#### 3.3.3 Happiness

Five items, based on the UN World Happiness Report framework (Helliwell et al., 2022), assessed life satisfaction, daily happiness, anxiety and depression (reverse-coded), and life meaning. Responses were given on a 10-point scale (1 = not at all to 10 = very much), and the mean score represented overall happiness.

#### 3.3.4 Cultural orientation

Countries were categorized using Gelfand et al. (2011) and Minkov and Kaasa (2022). South Korea and Japan were classified as collectivist-tight cultures; Germany and Finland as individualist-loose. This typology provided a theoretical basis for cross-cultural comparison (Table 2).

**Table 2:** Measurement instrument.

Variable	Sample item	Scale	Source
Stress of situational strength	“I often experience interference or control from my organization when I act according to my own values or beliefs.”	1 = Strongly disagree → 5 = Strongly agree	Leifels and Bowen (2021), Cohen and Wills (1985)
	“I feel stressed due to a sociocultural environment that conflicts with my personal values and opinions.”		
	“I feel pressure and control to constantly develop new skills and abilities.”		
	“I feel stressed about having to meet the skill and ability expectations imposed by society.”		
Self-efficacy	“I believe I can perform tasks better than others.” (confidence)	1 = Strongly disagree → 5 = Strongly agree	Dweck and Leggett (1988), Bandura and Cervone (1986), Locke and Latham (1990)
	“I enjoy taking on tasks that are more challenging than what most people usually do.” (preference for moderately difficult tasks)		
	“When faced with difficult situations, I remain composed and try to find solutions.” (self-regulation)		
Happiness	“Overall, how satisfied are you with your life these days?”	1 = Not at all → 10 = Very much	Helliwell et al. (2022), OECD (2022)
	“Did you feel happy yesterday?”		
	“Did you feel worried yesterday?” *(reversed)*		
	“Did you feel depressed yesterday?” *(reversed)*		
	“Do you feel that the things you do in your life are worthwhile?”		

To ensure the validity and reliability of the measurement instruments across cultures, both exploratory factor analysis (EFA) and multi-group confirmatory factor analysis (MGCFA) were conducted. Table 3 shows the measurement reliability and validity of the constructs used in the study. As shown in Table 3, all factor loadings exceeded 0.65, indicating acceptable construct validity. Internal consistency was also adequate for exploratory research, particularly given the conceptual breadth of the constructs and the cultural diversity of the sample (Taber, 2018; Schmitt, 1996).

**Table 3:** Measurement reliability and validity.

Variables	Items	Component			Total variance explained	Cronbach’s α	McDonald’s ω	CR
		1	2	3				
Stress of situational strength	Organizational Control Stress (R)	0.251	0.637	−0.001	Eigen value: 2.132 Variance %: 17.764 Cumulative %: 38.248	0.699	0.75	0.73
	Social Atmosphere Stress (R)	0.170	0.633	−0.016				
	Pressure and Control from New Technology (R)	0.040	0.765	0.085				
	Ability Strength (R)	0.102	0.789	0.157				
Self-efficacy	Confidence	0.048	0.042	0.734	Eigen value: 1.960 Variance %: 16.330 Cumulative %: 54.579	0.656	0.66	0.66
	Preference for Difficult Tasks	0.079	0.036	0.776				
	Self-Regulatory Efficacy	0.183	0.095	0.694				
Happiness	Overall Satisfaction with Life	0.693	0.141	0.358	Eigen value: 2.458 Variance %: 20.484 Cumulative %: 20.484	0.749	0.76	0.74
	Happiness Felt the Previous Day	0.747	0.160	0.223				
	Concern and Worry the Previous Day (R)	0.587	0.184	−0.227				
	Depression Felt the Previous Day (R)	0.754	0.144	0.005				
	Overall Value in Daily Activities	0.602	0.079	0.276				

KMO = 0.791; *x*^2^ = 1,713.121; df = 66; *p* < 0.001.

Principal axis factoring was used with varimax rotation and Kaiser normalization, *N* = 608.

Rotation converged into five iterations.

(R): reversed.

Measurement invariance was assessed using MGCFA. The configural model demonstrated acceptable fit (χ^2^ (204) = 407.55, RMSEA = 0.081, CFI = 0.857). Partial scalar invariance was supported, with the final model showing modest but acceptable fit indices (CFI = 0.711, RMSEA = 0.104). Although the CFI value was below conventional thresholds, partial invariance permits valid latent mean comparisons when at least two items—one anchor item and one additional invariant item—are consistent across groups (Jang et al., 2017; Byrne et al., 1989; Steenkamp and Baumgartner, 1998). Accordingly, the current model meets the minimum criteria for partial scalar invariance and supports cross-cultural latent mean comparisons within the proposed theoretical framework.

### 3.4 Analysis plan

Descriptive statistics and hierarchical regression analyses were conducted using IBM SPSS Statistics (Version 26.0). Multi-group confirmatory factor analysis (MGCFA) and bootstrapped structural equation modeling (SEM) were performed using the *lavaan* package in R (Version 4.5.0).

To examine whether cultural differences (individualism vs. collectivism) are associated with differences in situational strength stress, self-efficacy, and happiness, independent samples t-tests were conducted.

Hierarchical regression analysis to test the hypotheses was conducted in three steps. In the first step, control variables were entered, including marital status (0 = single; 1 = married/widowed/divorced), gender (0 = male; 1 = female), employment status (0 = employed; 1 = unemployed), age group (1 = 20–29 to 6 = 70+), education level (1 = middle school to 5 = graduate school), and income level (1 = lowest to 6 = highest). Cultural orientation was also included as a binary variable (0 = collectivist; 1 = individualist). In the second step, situational strength stress was added as the primary independent variable. In the third step, self-efficacy was included to assess its potential mediating role in the relationship between situational strength stress and happiness.

To further validate the mediation hypothesis, SEM was conducted using three latent variables—situational strength stress, self-efficacy, and happiness. Bootstrapping with 5,000 resamples was applied to estimate confidence intervals for indirect effects. Model fit was evaluated using standard indices: chi-square (χ^2^), comparative fit index (CFI), root mean square error of approximation (RMSEA), and standardized root mean square residual (SRMR). Mediation was supported based on the statistical significance of the indirect effect (a × b) and changes in the direct effect (c′) following the inclusion of the mediator.

## 4 Analytical results

### 4.1 Differences between individualist and collectivist cultures

#### 4.1.1 Stress of situational strength

When cohesion is weak between a collective and its components, organizations and societies normatively enforce various forms of control to integrate individuals into the group. This control tends to heighten perceptions of stress arising from situational strength, particularly when individuals perceive significant misalignment between personal values and those of society or the organization.

As shown in Table 4, this study revealed that individualist cultures, such as those found in Finland and Germany, reported relatively lower levels of stress associated with situational strength (*M* = 2.59, SD = 0.87). In contrast, collectivist cultures, such as those of South Korea and Japan, exhibited significantly higher levels of situational strength stress (*M* = 3.12, SD = 0.81). These findings suggest that in individualist cultures, where there is greater alignment between individual and organizational values, the stress caused by situational strength is comparatively lower.

**Table 4:** Differences in stress, self-efficacy, and happiness by cultural types and countries.

Variable	Culture type	Country	Mean	SD	Mean	SD	T-stat	*p*
Stress of situational strength	Collectivist culture	South Korea	3.22	0.79	3.12	0.81	7.716	0.000
		Japan	2.92	0.83				
	Individualist culture	Finland	2.56	0.76	2.59	0.87		
		Germany	2.62	0.95				
Self-efficacy	Collectivist culture	South Korea	3.58	0.69	3.50	0.72	−4.262	0.000
		Japan	3.35	0.73				
	Individualist culture	Finland	3.68	0.68	3.74	0.67		
		Germany	3.80	0.67				
Happiness	Collectivist culture	South Korea	6.93	1.82	6.76	1.90	−4.291	0.000
		Japan	6.43	2.01				
	Individualist culture	Finland	8.38	1.23	8.03	1.50		
		Germany	7.70	1.65				

In Finland and Germany, stress caused by societal pressures to align with societal norms (*M* = 2.94, SD = 1.23) and the pressure to acquire new skills and technologies (*M* = 2.92, SD = 1.31) were notably higher, while the lowest reported stress was related to organizational control (*M* = 1.96, SD = 1.18). In contrast, in South Korea and Japan, the highest levels of stress were attributed to pressures to acquire new skills and technologies (*M* = 3.27, SD = 1.11), followed closely by societal stress arising from differences in values (*M* = 3.14, SD = 1.13).

#### 4.1.2 Self-efficacy

As shown in Table 4, this study examined differences in self-efficacy between individualist and collectivist cultures. Self-efficacy is defined as an individual’s belief in their capacity to achieve desired outcomes and effectively manage challenges. Participants from individualist cultures—Finland and Germany—reported higher levels of self-efficacy (*M* = 3.74, SD = 0.67) than those from collectivist cultures—South Korea and Japan (*M* = 3.50, SD = 0.72).

Among the four countries, Germany exhibited the highest average level of self-efficacy (*M* = 3.80, SD = 0.67), followed by Finland (*M* = 3.68, SD = 0.68), South Korea (*M* = 3.58, SD = 0.69), and Japan (*M* = 3.35, SD = 0.73).

#### 4.1.3 Happiness

As illustrated in Table 4, individuals in individualist cultures—Finland and Germany—reported higher overall happiness (*M* = 6.39, SD = 1.03) compared to those in collectivist cultures—South Korea and Japan (*M* = 6.14, SD = 0.99).

Among the four countries, the highest levels of happiness were reported in Finland (*M* = 6.41, SD = 0.82), followed by Germany (*M* = 6.36, SD = 1.19), South Korea (*M* = 6.21, SD = 0.95), and Japan (*M* = 6.01, SD = 1.05).

### 4.2 Contributors to differences between individualist and collectivist cultures

As shown in Table 5, this study used t-tests to statistically verify the differences between individualist and collectivist cultures regarding situational strength stress, self-efficacy levels, and happiness.

**Table 5:** T-test results for stress, self-efficacy, and happiness by cultural types.

Variable		Culture type (*N*)	Mean	SD	T-stat	*p*
Stress of situational strength	Organizational control stress	Collectivist (294)	2.74	1.13	8.390***	0.000
		Individualist (314)	1.96	1.18		
	Societal atmosphere stress	Collectivist (294)	3.14	1.13	2.015*	0.044
		Individualist (314)	2.94	1.23		
	Pressure from new technology and control stress	Collectivist (294)	3.27	1.11	3.555***	0.000
		Individualist (314)	2.92	1.31		
	Stress related to abilities	Collectivist (294)	3.32	1.08	8.231***	0.000
		Individualist (314)	2.54	1.23		
Self-efficacy	Confidence	Collectivist (294)	3.59	0.89	−0.146	0.884
		Individualist (314)	3.60	0.88		
	Preference for challenging tasks	Collectivist (294)	3.32	0.98	−4.097***	0.000
		Individualist (314)	3.64	0.97		
	Self-control	Collectivist (294)	3.60	0.83	−5.628***	0.000
		Individualist (314)	3.98	0.86		
Happiness	Overall satisfaction with life	Collectivist (294)	6.76	1.90	−9.211***	0.000
		Individualist (314)	8.03	1.50		
	Happiness felt the previous day	Collectivist (294)	7.11	2.04	−5.354***	0.000
		Individualist (314)	7.99	2.02		
	Worry and concern the previous day	Collectivist (294)	5.57	2.43	0.064	0.949
		Individualist (314)	5.55	2.91		
	Depression felt the previous day	Collectivist (294)	4.07	2.49	6.941***	0.000
		Individualist (314)	2.68	2.44		
	Perception of meaningful work	Collectivist (294)	7.21	1.87	−2.808**	0.005
		Individualist (314)	7.68	2.26		

*p*: *< 0.05, **< 0.01, ***< 0.001.

A summary of the test results is as follows:

1. Situational strength stress imposed by organizations and societies demonstrated statistically significant differences between individualist and collectivist cultures. Specifically, organizational control stress (*t* = 8.390, *p* < 0.001), societal atmosphere stress (*t* = 2.015, *p* < 0.05), pressure from new technology and control stress (*t* = 3.555, *p* < 0.001), and stress related to abilities (*t* = 8.231, *p* < 0.001) were all significantly higher in collectivist cultures than individualist cultures.
2. Self-efficacy levels also showed significant differences between individualist and collectivist cultures. The preference for challenging tasks (*t* = −4.097, *p* < 0.001) and self-control (*t* = −5.628, *p* < 0.001) were significantly higher in individualist cultures compared to collectivist cultures. However, there was no statistically significant difference in confidence levels between the two cultures (*t* = −0.146, *p* = 0.884).
3. Happiness similarly exhibited significant differences between individualist and collectivist cultures, also detailed in Table 5. Specifically, overall life satisfaction (*t* = −9.211, *p* < 0.001), happiness felt the previous day (*t* = −5.354, *p* < 0.001), depression felt the previous day (*t* = 6.941, *p* < 0.001), and perceptions of meaningful work (*t* = −2.808, *p* < 0.005) were all significantly higher in individualist cultures compared to collectivist cultures. However, no significant difference was found in the level of worry or concern felt the previous day (*t* = 0.064, *p* = 0.949) between the two cultural orientations.

### 4.3 Results from hierarchical regression analyses

Table 6 shows the summary statistics used in the hierarchical analyses: 41.6% of respondents were married/widowed/divorced and 58.4% were single; 55.1% were females, and 44.9% were male; 22.2% were unemployed. The mean age group was 2.43, and the income group was 2.67; the mean education level was 3.38; 51.6% of respondents were from the individualist culture and 48.4% were from collectivist culture. The mean composite score for situational strength was 2.84; the mean composite score for self-efficacy was 3.62; the mean composite score for happiness was 6.12.

**Table 6:** Summary statistics for variables used in hierarchical analyses.

Variable	Mean	Std. dev.	Min.	Max.
Married	0.4161183	0.4933195	0	1
Female	0.5509868	0.4978031	0	1
Unemployed	0.2220395	0.4159598	0	1
Age	2.427632	1.422817	1	6
Income	2.669408	0.353835	1	6
Education	3.383224	1.065954	1	5
Individualist culture	0.516447	0.5001409	0	1
Situational strength	2.843755	0.8803233	1	5
Self-efficacy	3.625066	0.7030627	1.67	5
Happiness	6.120724	0.9647313	3.4	7.6

We employed a hierarchical regression model to test the hypotheses regarding the effects of situational strength stress on self-efficacy and happiness. The results of the empirical analysis are shown in Table 7.

**Table 7:** Results of hierarchical regression analyses (*N* = 608).

Model	Variable	*B*	SE	*β*	t(p)	F(p)	*R^2^*	adj. *R^2^*
Model 1 (Stress of situational strength ➔ Self-efficacy)	Constant	3.093	0.240		12.883***	10.987***	0.128	0.116
	Situational Stress	−0.083	0.033	−0.104	−2.469*			
	Individualist Culture	0.201	0.062	0.143	3.265**			
	Female	−0.132	0.057	−0.094	−2.338*			
	Age	−0.026	0.028	−0.052	−0.938			
	Married	0.157	0.077	0.110	2.035*			
	Unemployed	−0.036	0.069	−0.021	−0.522			
	Education	0.121	0.026	0.184	4.607***			
	Monthly Income	0.054	0.024	0.103	2.246*			
Model 2 (Stress of situational strength➔ Happiness)	Constant	6.260	0.328		19.112***	11.977***	0.138	0.126
	Situational Stress	−0.306	0.046	−0.279	−6.690***			
	Individualist Culture	0.107	0.084	0.055	1.275			
	Female	0.273	0.077	0.141	3.541***			
	Age	−0.030	0.038	−0.045	−0.807			
	Married	0.142	0.105	0.073	1.355			
	Unemployed	−0.054	0.095	−0.023	−0.569			
	Education	−0.035	0.036	−0.039	−0.982			
	Monthly Income	0.075	0.032	0.106	2.317*			
Model 3 (Stress of situational strength ➔ Happiness; Self-efficacy ➔ Happiness)	Constant	5.779	0.368		15.702***	7.868**	0.149	0.136
	Situational Stress	−0.293	0.046	−0.267	−6.413***			
	Self-Efficacy	0.155	0.055	0.113	2.805**			
	Individualist Culture	0.076	0.084	0.039	0.900			
	Female	0.294	0.077	0.152	3.812***			
	Age	−0.026	0.037	−0.039	−0.703			
	Married	−0.118	0.105	−0.060	1.125			
	Unemployed	−0.048	0.094	−0.021	−0.513			
	Education	−0.054	0.036	−0.060	−1.489			
	Monthly Income	0.067	0.032	0.094	2.064*			

*p*: * < 0.05, ** < 0.01, *** < 0.001.

#### 4.3.1 Regression results

In Model 1, the regression coefficient for the independent variable, situational strength stress, was *B* = −0.083 (*p* < 0.05). The model was statistically significant (*F* = 6.317, *p* < 0.001) and explained 12.8% of the variance in self-efficacy, with an adjusted R^2^ of 0.106. This result supports Hypothesis 1 (H1), indicating that situational strength stress negatively associated with self-efficacy.

In Model 2, the effect of situational strength stress on happiness was stronger (*B* = −0.306, *p* < 0.001). The model was significant (*F* = 6.936, *p* < 0.001) and explained 13.8% of the variance in happiness (adjusted *R*^2^ = 0.117). This supports Hypothesis 2 (H2), higher situational stress is significantly associated with lower happiness.

In Model 3, both situational strength stress (*B* = −0.293, p < 0.001) and self-efficacy (*B* = 0.155, *p* < 0.005) were significant predictors of happiness. The overall model remained significant (*F* = 7.001, *p* < 0.001), with a slightly improved adjusted *R*^2^ of 0.127. The decrease in the coefficient for situational stress—from *B* = −0.306 to B = −0.293—suggests a possible mediating role of self-efficacy. Additionally, the bootstrapped indirect effect was statistically significant (95% CI [−0.053, −0.013], *p* = 0.002), providing statistical support for Hypothesis 3 (H3).

**Figure 2:**
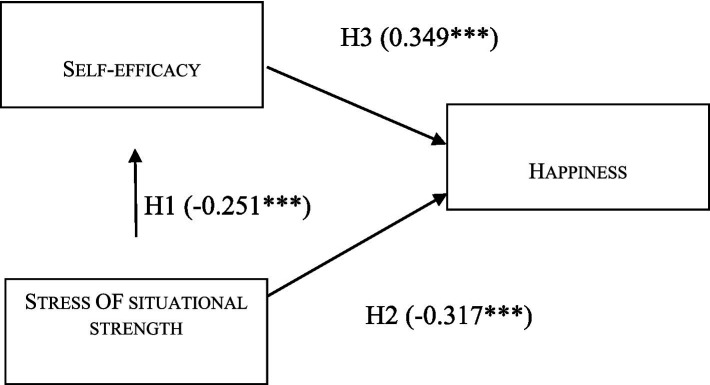
Results of analytical model. *p*: *< 0.05, **< 0.01, ***< 0.001.

To examine the hypothesized mediation effect of self-efficacy in the relationship between perceived situational strength and happiness, a structural equation model (SEM) with bootstrapped confidence intervals was tested using the total sample (N = 608). The overall model demonstrated acceptable fit to the data: *χ*^2^(51) = 223.89, *p* < 0.001; CFI = 0.873; RMSEA = 0.075 (90% CI [0.065, 0.085]); SRMR = 0.058 (Figure 2).

The path from perceived situational strength to self-efficacy was significant (*a* = −0.251, *p* < 0.001), as was the path from self-efficacy to happiness (*b* = 0.349, *p* < 0.001). The direct path from situational strength to happiness also remained significant (*c*′ = −0.317, *p* < 0.001). The indirect effect of situational strength on happiness via self-efficacy was statistically significant (indirect = −0.088, 95% CI [−0.053, −0.013], *p* = 0.002), suggesting partial mediation. The standardized indirect effect (*β* = −0.088) was modest but meaningful. The total effect was −0.405 (*p* < 0.001) (Table 8).

**Table 8:** Mediation analysis results.

Path	Unstandardized estimate	Standardized estimate	Std. Error	*z-*value	*p*-value	CI Lower	CI Upper
Stress of situational strength→ self-efficacy	−0.207	−0.251	0.053	−3.929	< 0.001	−0.313	−0.108
self-efficacy → happiness	0.143	0.349	0.034	4.217	< 0.001	0.081	0.212
Stress of situational strength → happiness	−0.107	−0.317	0.021	−5.022	< 0.001	−0.152	−0.069
indirect (a*b)	−0.03	−0.088	0.010	−3.042	0.002	−0.053	−0.013
direct (c’)	−0.107	−0.317	0.021	−5.02	< 0.001	−0.152	−0.069
Total	−0.136	−0.405	0.023	−5.968	< 0.001	−0.183	−0.097

These results suggest that stronger situational constraints are associated with lower self-efficacy, which in turn is related to decreased happiness.

#### 4.3.2 Notable results from control variables

The regression models also revealed meaningful patterns across the control variables. Gender consistently emerged as a significant predictor across all three models. Specifically, being female was associated with a significantly lower level of self-efficacy (Model 1: *B* = −0.132, *p* < 0.05), yet with notably higher levels of happiness (Model 2: *B* = 0.273, *p* < 0.001; Model 3: B = 0.294, *p* < 0.001).

Age, in contrast, did not yield significant effects in any of the models (Model 1: *B* = −0.021, *p* = 0.612; Model 2: *B* = 0.008, *p* = 0.838; Model 3: *B* = 0.012, *p* = 0.786). These null results imply that within the age brackets assessed in this study, chronological age may not substantially influence either self-efficacy or happiness.

Similarly, marital status, which was operationalized as a binary indicator of being currently unmarried (including single, divorced, or widowed), was not significantly associated with either outcome variable. The coefficients remained nonsignificant in all models (Model 1: *B* = 0.044, *p* = 0.559; Model 2: *B* = 0.072, *p* = 0.347; Model 3: *B* = 0.068, *p* = 0.371), suggesting that the mere presence or absence of a marital relationship may not play a decisive role in shaping psychological well-being—particularly in the context of societies where non-traditional family structures are increasingly normalized.

Employment status, however, displayed consistently negative effects. Being unemployed significantly reduced both self-efficacy (Model 1: *B* = −0.161, *p* < 0.05) and happiness (Model 2: *B* = −0.253, *p* < 0.01; Model 3: *B* = −0.239, *p* < 0.01), reaffirming the well-documented psychological costs of unemployment and role loss.

Educational attainment was only marginally significant in predicting self-efficacy (Model 1: *B* = 0.087, *p* < 0.10), with no significant influence on happiness (Model 2: *B* = 0.029, *p* = 0.621; Model 3: *B* = 0.024, *p* = 0.678). This pattern suggests that education may enhance confidence in one’s abilities but does not directly translate into greater subjective well-being.

Monthly income, by contrast, was a robust predictor of happiness. In both Models 2 and 3, higher income levels were significantly associated with higher happiness (Model 2: *B* = 0.174, *p* < 0.01; Model 3: *B* = 0.165, *p* < 0.01), although no significant effect was found on self-efficacy (Model 1: *B* = 0.051, *p* = 0.307).

Lastly, cultural orientation yielded positive effects across both outcomes. Respondents from individualist cultures reported marginally higher self-efficacy (Model 1: *B* = 0.094, *p* < 0.10) and significantly greater happiness (Model 2: *B* = 0.228, *p* < 0.01; Model 3: *B* = 0.217, *p* < 0.01). These findings are consistent with existing literature suggesting that individualist values—emphasizing autonomy and personal agency—may enhance psychological well-being.

Taken together, these results demonstrate that gender, employment, income, and cultural orientation are meaningful predictors of self-efficacy and happiness; age, marital status, and education appear to exert limited or inconsistent influence in this cross-cultural context.

## 5 Discussion

### 5.1 Implications for future research

This study examined how situational strength influences self-efficacy and happiness across four cultural contexts—South Korea, Japan, Finland, and Germany—each representing varying degrees of collectivism and cultural tightness. The findings demonstrate that individuals in collectivist and tight cultures experience higher stress from situational strength, particularly related to societal expectations regarding abilities and roles. For instance, South Korea, despite its Confucian similarities with Japan and China, exhibits a uniquely strict adherence to social norms, reinforcing intense achievement pressure and social conformity (Gelfand et al., 2011).

Interestingly, Finland, although generally categorized as individualist, reported the highest levels of stress from societal pressure to conform. In contrast, participants from individualist and loose cultures such as Germany and Finland reported lower situational stress, greater perceived autonomy, and higher happiness levels. These results align with the 2022 World Happiness Report (Helliwell et al., 2022), which ranks individualist cultures higher in subjective well-being.

While both Japan and South Korea are commonly classified as collectivist cultures, their psychological responses diverged meaningfully. Japan’s relatively low self-efficacy may reflect long-standing cultural values emphasizing modesty, self-restraint, and relational harmony (Davies and Ikeno, 2011), whereas South Korea’s emphasis on high achievement and status-based competition fosters a distinct psychological climate. These findings highlight the importance of disaggregating collectivist cultures—especially in East Asia—where shared classifications can obscure meaningful internal differences in cultural norms and individual behavior.

The negative impact of situational strength on psychological outcomes also varied. While both self-efficacy and happiness were adversely affected, the association was stronger for happiness. This suggests that normative pressure may have more immediate emotional effects than motivational ones. Self-efficacy partially mediated the relationship between situational strength and happiness, but the modest reduction in the direct effect implies the presence of additional mediating mechanisms, such as resilience, coping strategies, or social support (Bandura, 1997; Luthans and Youssef, 2004).

Importantly, in collectivist cultures, self-efficacy is not primarily driven by intrinsic motivation but is reinforced through group recognition and social validation (Bandura, 1997; Loton and Waters, 2017; Schunk and Pajares, 2005). When individual values and competencies misalign with organizational or societal expectations, the resulting situational strength manifests as greater institutional control over roles, tasks, and decision-making (French and Caplan, 1972; Karasek and Theorell, 1990), which contributes to elevated stress and diminished self-efficacy (Chen et al., 2016).

Moreover, situational strength is not a unitary construct; it is comprised of four subdimensions—clarity, consistency, constraints, and consequences (Meyer et al., 2010). While this study examined situational strength in a general sense, future research should explore how each subdimension differentially influences psychological outcomes across cultural contexts. For example, constraints and consequences may have stronger negative impacts in tight, collectivist societies where rule adherence is strictly enforced, whereas clarity may be less burdensome in high-context cultures. Disentangling these dimensions could yield richer insights into the specific pathways through which cultural norms shape psychological stress and adaptation.

Cross-cultural differences may also influence the validity of self-report measures, as situational strength, self-efficacy, and happiness are culturally interpreted constructs (Ajiboye and Olubela, 2020; Diener and Diener, 1995). Gelfand and Lun (2013) emphasized that situational strength is embedded within broader historical and ecological contexts. Accordingly, scholars increasingly advocate for the use of measurement equivalence models when conducting international comparisons, acknowledging partial invariance as sufficient for meaningful interpretation (Dalal et al., 2020; Meyer et al., 2010).

In terms of control variables, gender and employment status consistently predicted psychological outcomes. Women reported higher happiness but lower self-efficacy, potentially reflecting culturally shaped emotional processing and gender role expectations. Unemployment predicted lower scores on both self-efficacy and happiness, underscoring the psychological importance of occupational identity. Meanwhile, age and marital status had no significant effects, suggesting that cultural and structural factors may overshadow individual or generational differences in influencing psychological well-being, particularly in collectivist societies where relational roles are deeply embedded (Oishi et al., 1999; Suh et al., 1998).

This study extends the situational strength framework by applying it to non-Western, collectivist societies and shifting the analytic focus from institutional compliance to subjective well-being. While previous research has emphasized the role of situational strength in predicting performance and conformity within organizations (e.g., Meyer et al., 2010), the current study highlights its broader psychological relevance across cultures. The findings call for culturally nuanced models that incorporate the interaction between situational environments and internal psychological resources (Hong and Yeo, 2022).

Future studies should further explore these dynamics using longitudinal designs and additional mediators, such as institutional trust, emotion regulation, and social capital. Incorporating the multidimensionality of situational strength and refining cultural distinctions—particularly within broader cultural categories such as East Asia—will enhance the theoretical sophistication and cross-cultural relevance of future research in this domain.

### 5.2 Study limitations

While this study offers valuable cross-cultural insights, several methodological limitations should be acknowledged.

Data were collected from 608 urban participants in Seoul, Tokyo, Helsinki, and Hamburg. While suitable for cross-national comparison, this urban sampling limits generalizability to rural populations, where social norms may differ significantly—particularly in collectivist societies. Older adults (60+) were underrepresented in South Korea and Japan, restricting analysis of generational differences. Marital status was coded as a binary variable, which may not reflect the diversity of family structures in Finland and Germany.

Lastly, the use of a cross-sectional design limits the ability to infer causality. Although the model assumes that self-efficacy mediates the relationship between situational stress and happiness, it is also plausible that situational strength is influenced by individuals’ perceived self-efficacy. Future studies employing longitudinal or experimental designs could help clarify the directionality of these relationships.

## 6 Conclusion

This study demonstrates the significant psychological implications of situational strength on self-efficacy and happiness across diverse cultural contexts, particularly along the dimensions of individualism–collectivism and cultural tightness–looseness. The findings confirm that cultural context critically shapes how individuals perceive and respond to situational demands, especially those related to normative expectations and societal control (Gelfand et al., 2011; Oishi et al., 1999).

In collectivist and tight cultures, individuals tend to experience stronger situational strength due to heightened pressures to conform to group norms. This elevated normative control increases stress and undermines self-efficacy, leading to diminished happiness. These results expand upon Meyer et al.'s (2010) conceptualization of situational strength as an organizational construct by demonstrating its broader psychological effects across societal contexts. Contrary to earlier assumptions that collectivism enhances collective or social efficacy (Bandura, 1997), the current findings suggest that when situational strength becomes excessive, it may suppress rather than support individual agency and emotional outcomes like happiness.

This pattern may be explained by cultural mechanisms such as the internalization of group-based evaluation standards (Schunk and Pajares, 2005), which in turn weaken self-determined motivation. In contrast, individualist and loose cultures foster autonomy and flexibility, which buffer the psychological burden associated with social expectations and enhance both self-efficacy and happiness (Helliwell et al., 2022).

The study contributes to theoretical development by extending the scope of situational strength research beyond organizational performance to consider emotional and motivational outcomes such as happiness. It also supports recent calls to apply more culturally responsive approaches to cross-cultural psychology and comparative stress research (Dalal et al., 2020; Gelfand and Lun, 2013). By disaggregating collectivist cultures and emphasizing intraregional variation—particularly within East Asia—the findings challenge overly homogenized cultural classifications and offer a more nuanced understanding of how cultural norms influence psychological adaptation.

In terms of practical implications, the results suggest that reducing excessive normative control and enhancing personal autonomy could contribute to improved happiness in collectivist societies. In contexts like South Korea, where societal pressures remain high, such interventions may be particularly valuable. These policy insights may be extended to other culturally tight societies undergoing modernization.

Future research should adopt longitudinal or experimental designs to better establish causal mechanisms. Investigating mediators such as emotion regulation, institutional trust, or social capital may further illuminate how situational strength translates into psychological outcomes. Moreover, exploring the distinct subdimensions of situational strength—clarity, consistency, constraints, and consequences—could deepen our understanding of how environmental factors interact with cultural systems to shape happiness.

## Data Availability

The datasets presented in this article are not readily available because the National Research Foundation of Korea has the right of the data. Requests to access the datasets should be directed to ejuho79@gmail.com.
