# Supplementary material for: A study of the effects of situational strength on self-efficacy and happiness: comparing individualist and collectivist cultures
Source: Front Psychol. 2025 Jun 27;16:1563643. doi: 10.3389/fpsyg.2025.1563643 (PMC12245773; doi:10.3389/fpsyg.2025.1563643)
Supplement: Supplementary file 1 [file Supplementary_file_1.docx]

Appendix: Survey Items by Country

[Korea]

| Variable | Survey Item | Scale |
| --- | --- | --- |
| Stress of situational strength | 내 가치, 생각에 따른 행동이나 태도로 조직에서 간섭이나 통제를 받는 편이다. | 1 = 전혀 아니다 ~ 5 = 매우 그렇다 |
|  | 나는 가치관이나 생각과 다른 사회분위기로 스트레스를 받는다. |  |
|  | 새로운 능력과 기술을 갖춰야하는 압박과 통제를 느낀다. |  |
|  | 사회가 요구하는 능력을 갖추려면 스트레스가 생긴다. |  |
| Self-efficacy | 나는 다른 사람들보다 일을 더 잘할 수 있다. | 1 = 전혀 아니다 ~ 5 = 매우 그렇다 |
|  | 나는 누구나 하는 일보다 다소 난이도 있는 일에 도전하는 것을 즐긴다. |  |
|  | 나는 어려운 일에 직면해도 침착하게 대처하며 해결방법을 찾는다. |  |
| Happiness | 귀하는 요즘 자신의 삶에 대하여 전반적으로 만족한다고 생각하십니까? | 1 = 전혀 아니다 ~ 10 = 매우 그렇다 |
|  | 귀하는 어제 행복했다고 생각하십니까? |  |
|  | 귀하는 어제 근심과 걱정이 있었습니까? |  |
|  | 귀하는 어제 우울했습니까? |  |
|  | 귀하가 요즘 하는 일은 전반적으로 가치 있는 일이라고 생각하십니까? |  |

[Germany]

| Variable | Survey Item | Scale |
| --- | --- | --- |
| Stress of situational strength | \|  \| \| --- \|  \| Ich habe das Gefühl, dass ich aufgrund meiner Werte oder Überzeugungen von meiner Organisation kontrolliert oder eingeschränkt werde. \| \| --- \| | 1 = Trifft gar nicht zu ~ 5 = Trifft völlig zu |
|  | Ich empfinde Stress aufgrund gesellschaftlicher Stimmungen, die nicht mit meinen Werten oder Überzeugungen übereinstimmen. |  |
|  | Ich fühle mich unter Druck gesetzt oder kontrolliert, neue Fähigkeiten und Kenntnisse zu erwerben. |  |
|  | Ich empfinde Stress dabei, die von der Gesellschaft geforderten Fähigkeiten zu erlangen. |  |
| Self-efficacy | Ich glaube, dass ich Aufgaben besser erledigen kann als andere. | 1 = Trifft gar nicht zu ~ 5 = Trifft völlig zu |
|  | Ich genieße es, mich Herausforderungen zu stellen, die schwieriger sind als das, was andere tun |  |
|  | Ich kann auch in schwierigen Situationen ruhig bleiben und Lösungen finden. |  |
| Happiness | Wie zufrieden sind Sie derzeit insgesamt mit Ihrem Leben? | 1 = Trifft gar nicht zu ~ 10 = Trifft völlig zu |
|  | Haben Sie sich gestern glücklich gefühlt? |  |
|  | Hatten Sie gestern Sorgen oder Bedenken? |  |
|  | Haben Sie sich gestern niedergeschlagen oder deprimiert gefühlt? |  |
|  | Haben Sie das Gefühl, dass das, was Sie derzeit tun, im Allgemeinen sinnvoll ist? |  |

[Finland]

| Variable | Survey Item | Scale |
| --- | --- | --- |
| Stress of situational strength | \|  \| \| --- \|  \| Koen, että organisaationi kontrolloi tai rajoittaa minua siksi, että toimin arvojeni ja ajatusteni mukaisesti. \| \| --- \| | 1 = Täysin eri mieltä ~ 5 = Täysin samaa mieltä |
|  | Koen stressiä yhteiskunnallisesta ilmapiiristä, joka ei vastaa omia arvojani tai ajatuksiani. |  |
|  | Koen painetta tai kontrollia hankkia uusia taitoja ja osaamista. |  |
|  | Koen stressiä pyrkiessäni saavuttamaan yhteiskunnan edellyttämiä taitoja. |  |
| Self-efficacy | Uskon, että suoriudun tehtävistä paremmin kuin muut. | 1 = Täysin eri mieltä ~ 5 = Täysin samaa mieltä |
|  | Nautin haasteellisemmista tehtävistä kuin mitä muut yleensä tekevät. |  |
|  | Pystyn säilyttämään rauhallisuuteni ja löytämään ratkaisuja myös vaikeissa tilanteissa. |  |
| Happiness | Kuinka tyytyväinen olet tällä hetkellä elämääsi kokonaisuutena? | 1 = Täysin eri mieltä ~ 10 = Täysin samaa mieltä |
|  | Tunsitko olosi onnelliseksi eilen? |  |
|  | \|  \| \| --- \|  \| Tunsitko eilen huolta tai murhetta? \| \| --- \| |  |
|  | Tunsitko eilen alakuloisuutta tai masennusta? |  |
|  | Koetko, että nykyinen tekemisesi on yleisesti ottaen merkityksellistä? |  |

[Japan]

| Variable | Survey Item | Scale |
| --- | --- | --- |
| Stress of situational strength | 自分の価値観や考えに基づいた行動や態度のために、組織から干渉や統制を受けていると感じる。 | 1 = 全くそう思わない ~ 5 = 非常にそう思う |
|  | 自分の価値観や考えと異なる社会的な雰囲気によってストレスを感じる。 |  |
|  | 新しいスキルや知識を身につけなければならないというプレッシャーや統制を感じる。 |  |
|  | 社会から求められる能力を身につけることにストレスを感じる。 |  |
| Self-efficacy | 自分は他の人よりもうまく仕事ができると思う。 | 1 = 全くそう思わない ~ 5 = 非常にそう思う |
|  | 誰もがする仕事よりも少し難しい課題に挑戦することを楽しむ。 |  |
|  | 困難な状況でも冷静に対応し、解決策を見つけることができる。 |  |
| Happiness | 最近のあなたの生活全体にどのくらい満足していますか？ | 1 = 全くそう思わない ~ 10 = 非常にそう思う |
|  | 昨日、幸せだと感じましたか？ |  |
|  | 昨日、不安や心配がありましたか？ |  |
|  | 昨日、憂鬱だと感じましたか？ |  |
|  | 最近取り組んでいることは、全体的に価値があると感じていますか？ |  |
